# A nephritic puzzle: C3-dominant glomerulonephritis as a sentinel of hidden autoinflammatory disease

**DOI:** 10.1007/s00467-026-07220-x

**Published:** 2026-02-20

**Authors:** Ryan C. Ward, Polly Ferguson, Prerna Rastogi, Carla Nester, Hua Sun

**Affiliations:** 1 Division of Nephrology, Stead Family Department of Pediatrics, Carver College of Medicine, The University of Iowa, Iowa City, USA; 2 Division of Rheumatology, Stead Family Department of Pediatrics, Carver College of Medicine, The University of Iowa, Iowa City, USA; 3 Department of Pathology, Carver College of Medicine, The University of Iowa, Iowa City, USA; 4 Iowa City, USA

**Keywords:** MPGN, Complement pathway, HIDS

## Abstract

Membranoproliferative glomerulonephritis (MPGN) is among the most challenging glomerulonephritides to diagnose and manage, given its clinico-immunopathologic heterogeneity. Although recent advances have introduced new therapies for complement-mediated and immune-complex MPGN, a substantial subset of cases remains poorly understood and lacks effective treatment. We report a pediatric case of biopsy-confirmed C3-dominant MPGN without evidence of complement dysregulation, with disease flares triggered by febrile episodes. Genetic testing identified underlying hyper-IgD syndrome (HIDS), a rare autoinflammatory disorder characterized by recurrent fevers and immune dysregulation. The patient’s MPGN was refractory to conventional immunosuppressive therapy but achieved remission with IL-1β blockade that targets HIDS. This case highlights the importance of considering alternative etiologies—including autoinflammatory diseases—in patients with MPGN when complement dysregulation is not evident.

## Background

Membranoproliferative glomerulonephritis (MPGN) is a chronic proliferative glomerulonephritis most often characterized by proteinuria, hematuria, and progressive loss of kidney function [1]. Idiopathic MPGN is now categorized into 1) complement-mediated MPGN, resulting from dysregulated complement system alternative pathway (AP) activation due to genetic mutations or autoantibodies, and 2) immune complex (IC)-mediated MPGN, where complement is not the primary driver and no underlying infection or autoimmune disease is identified [1, 2]. Many cases exist that have atypical features or poor response to classical immunosuppressant therapy, making diagnosis and treatment difficult. We report a case with histological features of C3-dominant MPGN, yet without evidence of AP dysfunction. Distinguishing between these subtypes is critical, as the underlying pathogenesis informs treatment decisions, particularly with the emergence of targeted complement therapies that may reduce the need for broad immunosuppression. For example, iptacopan, a selective Factor B inhibitor, is preferred when AP dysregulation drives disease (e.g., C3G), potentially preserving classical and lectin complement activity, whereas pegcetacoplan, a C3 inhibitor, offers broader complement suppression and may be used across both MPGN subtypes [1, 3].

## Case report

**Fig. 1:**
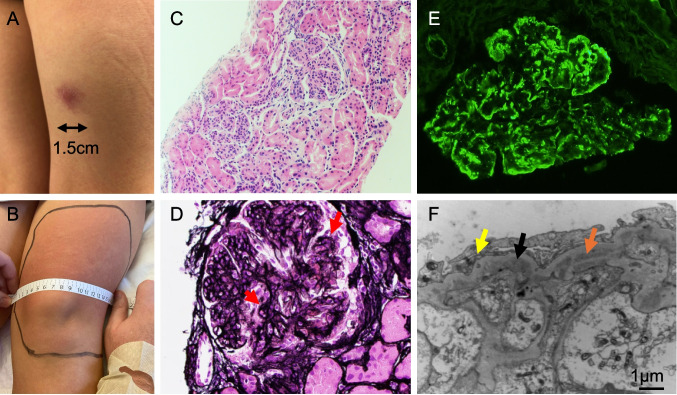
Clinico-pathological features **A**. Erythematous pustular skin lesion at the posterior thigh (**A**) and large erythematous rash in front of the knee (**B**). C-F: Kidney pathology. H&E (**C**) and Periodic Acid Schiff Methenamine Silver Stain (**D**) showed lobular mesangial expansion with rare double contour of the glomerular basement membrane (red arrows). **E**. C3 dominant staining. **F**. Transmission electron microscopy showed subendothelial (black arrow) and intramembranous (orange arrow) immune-complex type electron dense deposits. Moderate to severe podocyte foot process effacement (yellow arrow)

A 5-year-old girl was admitted for an acute kidney injury (serum creatinine [sCr] 0.6–1.6 mg/dL from a baseline of 0.4 mg/dL), hematuria (150 RBCs/HPF) and proteinuria (urine protein-to-creatinine ratio [UPC] 0.7–1.8 mg/mg) which had been noted during a febrile episode. She had experienced these recurrent febrile episodes since infancy, most often attributed to upper respiratory viral infections. It was unknown whether similar kidney injury had occurred during prior febrile episodes or whether chronic kidney disease was present beforehand. No history of urinary tract infections, strep throat, skin infections, or autoimmune symptoms (oral ulcers, joint pain, etc.) were reported. Family history was notable for scalp psoriasis in her father, but no known autoimmune or chronic kidney disease. On exam, she was well nourished and developed, euvolemic, but was hypertensive (130/84 mmHg). She had maculopapular skin lesions on the right elbow, posterior thigh, and knee (Fig. 1A, B), which family reported often appeared during illnesses.

Her laboratory work-up was notable for negative autoantibodies (ANA, anti-GBM, ANCA) and hepatitis B/C serologies, and normal C3 and C4. Kidney biopsy revealed C3-dominant glomerulonephritis [C3 (3 +), C1q (1 +), IgM (1 +), trace IgG], with mesangial expansion and capillary loop double contours, consistent with MPGN (Fig. 1C–E). Electron microscopy revealed subendothelial and intramembranous electron-dense deposits (Fig. 1F). Immunotyping electrophoresis showed no monoclonal immunoglobulins. Further evaluation for evidence of complement dysregulation was unrevealing: a C3G complement panel (MORL, University of Iowa) showed no autoantibodies to CFB, CFH, C3 convertase, or C5 convertase, and no evidence of AP dysregulation; a Natera Renasight panel revealed no pathogenic variants in common complement genes (*CFB, CFD, CFI, CFH*).

In the absence of evidence for complement dysregulation, the patient was initially treated for idiopathic MPGN per KDIGO guidelines, including steroids, mycophenolate mofetil for induction, and lisinopril for antihypertensive and antiproteinuric therapy. She achieved only a partial response (UPC 0.65 mg/mg) with recurrent glomerulonephritis flares during febrile illnesses. To improve disease control, she subsequently received six monthly cyclophosphamide doses followed by azathioprine monotherapy, which also failed to achieve sustained remission. Over the subsequent 17 months, she continued to have multiple flares associated with viral infections or fever-associated flares with systemic symptoms including abdominal, leg, and back pain, oral ulcers, and lethargy. During these flares, sCr and UPC rose to 0.8 mg/dL and 2.82 mg/mg, respectively.

Rheumatology evaluation was also pursued given flares of systemic symptoms (fevers, mucocutaneous lesions, limb pain) and elevated inflammatory markers (ESR, CRP, ferritin) which are not classically associated with kidney-limited MPGN. Common rheumatologic etiologies, including the juvenile idiopathic arthritis, periodic fever syndromes, familial Mediterranean fever, Castleman’s disease, systemic lupus erythematosus, and primary immunodeficiency, were considered. Notably, a markedly elevated IgD (211.7 mg/dL; normal < 15.3 mg/dL) provided evidence of immune dysregulation, raising concern for an underlying inborn error of immunity. A primary immunodeficiency genetic panel (INVITAE) revealed compound heterozygous pathogenic variants in the mevalonate kinase (MVK) gene: one point mutation that resulted in p.Val377Ile and a novel exon 3 deletion predicted to cause loss of function. A diagnosis of hyper-IgD syndrome (HIDS) was established. Canakinumab, a monoclonal antibody targeting IL-1β, was initiated. She has since remained free of febrile episodes and associated GN flares, with a stable UPC (0.3–0.4 mg/mg) and normalized sCr (0.4–0.5 mg/dL).

## Discussion

This case describes a pediatric patient with C3-dominant MPGN who demonstrated a poor response to standard immunosuppressive therapy with corticosteroids and mycophenolate mofetil. As accumulating evidence supports the efficacy of complement-targeting therapies in C3 glomerulopathy (C3G) [3, 4], these agents became increasingly attractive therapeutic options. However, in the absence of evidence for AP dysregulation in our patient, iptacopan—a selective Factor B inhibitor—was considered less likely to be effective. Pegcetacoplan, which provides broader inhibition of complement activation, appeared more mechanistically plausible. Nevertheless, the safety of complement-targeting therapies, particularly with respect to infection risk in the pediatric population, remains incompletely defined and represents a significant barrier to adoption in children, despite their therapeutic promise in adults.

Meanwhile, careful evaluation of systemic features beyond kidney-specific pathophysiology revealed HIDS-related autoinflammation as the underlying disease driver, allowing initiation of targeted anti–IL-1β therapy with improved clinical response. This case highlights both the diagnostic complexity of MPGN and the critical importance of identifying the primary pathogenic mechanism to guide therapy in an era of increasingly targeted treatments.

HIDS (OMIM #260,920) is a rare autosomal-recessive, autoinflammatory disorder caused by MVK deficiency [5]. Murine studies have shown that her genotype harbors only 9% of MVK activity, which can drop below 2% during fevers, exacerbating inflammatory flares [6]. Deficiency of this enzyme impairs prenylation of small GTPase proteins, resulting in Caspase-1 mediated IL-1β release and hyperinflammation [5]. Clinical features include fever, gastrointestinal discomfort, arthralgia, and skin lesions (such as actinic porokeratosis) with episodes triggered by infection, vaccination, or trauma [5, 7]. Canakinumab, the first FDA-approved biologic for HIDS, demonstrated efficacy in improving remission and reducing disease severity, with approximately 64% of patients remaining flare-free in clinical studies [8].

Kidney involvement in HIDS is rarely reported, with only one case of associated pauci-immune crescentic GN found in the literature [9]. Herein, we report the first case of C3-dominant MPGN likely triggered by the autoinflammation of HIDS. The precise mechanism linking HIDS to C3-dominant GN remains incompletely understood, but is supported by: 1) evidence that IL-1 signaling can induce or reciprocally dysregulate the AP pathway [10]; 2) clinical remission of our patient’s MPGN after IL-1β blockade with canakinumab. Further mechanistical investigation is needed to elucidate the causal effect of HIDS in MPGN with such C3-dominant deposits.

With complement inhibitors emerging for C3G, this case also underscores the importance of considering alternative etiologies, especially when AP dysregulation is absent, before pursuing these therapies. A broad differential diagnosis for such a case with obvious systemic symptoms is warranted and could include juvenile idiopathic arthritis, periodic fever syndromes, and lupus activation syndrome. Blood and urine biomarkers can aid in assessing underlying inflammation (CRP, ESR, ferritin) and immune dysregulation (autoantibodies, immunoglobulin levels and subclasses). In our case, elevated serum IgD and increased urine mevalonic acid, a substrate of MVK, can support the clinical diagnosis, and incorporation of MVK gene testing into kidney disease/C3G. MPGN diagnostic panels may help identify patients who could benefit from alternative targeted therapies.

## Summary

### What is new?

- C3-dominant MPGN may reflect underlying autoinflammatory disease, including hyper–IgD syndrome. In the absence of complement dysregulation, attention to extrarenal symptoms can guide mechanism-based targeted therapy and reduce reliance on broad immunosuppression.

## Data Availability

All original data supporting the findings of this case are provided in this report.
